# A PDA‐Functionalized 3D Lung Scaffold Bioplatform to Construct Complicated Breast Tumor Microenvironment for Anticancer Drug Screening and Immunotherapy

**DOI:** 10.1002/advs.202302855

**Published:** 2023-07-09

**Authors:** Wanheng Zhang, Yan Chen, Mengyuan Li, Shucheng Cao, Nana Wang, Yingjian Zhang, Yongtao Wang

**Affiliations:** ^1^ Shanghai Engineering Research Center of Organ Repair School of Medicine Shanghai University Shanghai 200444 China; ^2^ Department of Pharmacy The First Affiliated Hospital and College of Clinical Medicine of Henan University of Science and Technology Luoyang 471003 China; ^3^ School of Pharmacy Nanjing University of Chinese Medicine Nanjing 210023 China; ^4^ Department of Quantitative Life Sciences McGill University Montréal Québec H3A 0G4 Canada; ^5^ Department of Pediatrics Shanghai General Hospital Shanghai Jiao Tong University Shanghai 200080 China

**Keywords:** breast cancer cells, drug resistance screening, PDA‐modified lung scaffolds, tumor immunotherapy, underlying mechanism

## Abstract

2D cell culture occupies an important place in cancer progression and drug discovery research. However, it limitedly models the “true biology” of tumors in vivo. 3D tumor culture systems can better mimic tumor characteristics for anticancer drug discovery but still maintain great challenges. Herein, polydopamine (PDA)‐modified decellularized lung scaffolds are designed and can serve as a functional biosystem to study tumor progression and anticancer drug screening, as well as mimic the tumor microenvironment. PDA‐modified scaffolds with strong hydrophilicity and excellent cell compatibility can promote cell growth and proliferation. After 96 h treatment with 5‐FU, cisplatin, and DOX, higher survival rates in PDA‐modified scaffolds are observed compared to nonmodified scaffolds and 2D systems. The E‐cadhesion formation, HIF‐1α‐mediated senescence decrease, and tumor stemness enhancement can drive drug resistance and antitumor drug screening of breast cancer cells. Moreover, there is a higher survival rate of CD45^+^/CD3^+^/CD4^+^/CD8^+^ T cells in PDA‐modified scaffolds for potential cancer immunotherapy drug screening. This PDA‐modified tumor bioplatform will supply some promising information for studying tumor progression, overcoming tumor resistance, and screening tumor immunotherapy drugs.

## Graphical abstract

A 3D organotypic tumor biosystem is synthesized by polydopamine (PDA) modification of decellularized porcine lung tissues. PDA‐modified scaffolds present excellent water absorption, hydrophilia, and biocompatibility, as well as long‐term survival of CD45^+^/CD3^+^/CD4^+^/CD8^+^ T cells. The molecular mechanism of cell response to chemotherapeutic drugs is correlated with E‐cadherin, hypoxia, stemness, and reactive oxygen species removal. PDA‐modified scaffolds can provide a promising platform for immune‐oncology drug screening.

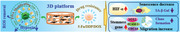

## 1 Introduction

Anticancer drug screening trials are generally inefficient in cancer biology and drug discovery due to the weak interaction between 2D tumor culture system and pathophysiological state of human, especially for immunotherapeutic drugs.^[^ ^1^ ^]^ Cancer immunotherapy has become a hot spot in cancer treatment research since it mainly uses cytotoxic T cell to specifically recognize and kill cancer cells compared to chemotherapy.^[^ ^2^ ^]^ It is vital to develop a constructed milieu that incorporates diverse immune and stromal cell populations to constitute the complex tumor microenvironment.^[^ ^3^ ^]^ However, the 2D cell culture system has some inevitable challenges in simulating the tumor microenvironment, such as cell monolayer limitation, insufficient network structures, and restricted spatial distribution. In contrast, 3D models may circumambulate these deficiencies and closely mimic complicated tumor microenvironment to show the advantages of biological responses between tumor cells and extracellular matrix (ECM), which has attracted increasing interest in cancer immune therapy.^[^ ^4^ ^]^

The 3D bioengineered tumor models based on advanced functional biomaterials are designed and established from natural materials or synthetic compounds through some distinctive techniques. Although the synthetic compounds including poly(caprolactone) (PCL), poly(lactic‐*co*‐glycolic acid) (PLGA), and poly(lactic acid) (PLA) are accessible to chemically decorate and mimic sophisticated tumor microenvironment, synthetic scaffolds show low cell adhesion ability and accidental loss of cell functions due to the deficiencies of inherent cell‐targeted attachment anchors.^[^ ^5^ ^]^ On the other hand, natural biomaterials primarily including collagen, alginate, and hyaluronic acid, enable to detour these adverse biological phenomena for efficient cell adhesion based on the inherent components and constructions.^[^ ^6^ ^]^ Nevertheless, natural‐component scaffolds were extremely limited in narrow clinical application owing to poor mechanical behavior.^[^ ^7^ ^]^ Decellularized matrices will supply a powerful apparatus to resemble tumor microenvironment via their excellent biomechanical characteristics and perfect ECM architectures. Gene expression patterns of tumor cells on lung scaffolds can show similar results with those of poor prognosis in tumor patients.^[^ ^8^ ^]^ A potential tumor engineering scaffold has been synthesized by photooxidative crosslinking reaction of decellularized tumor ECM to exhibit strong cell division ability, increased cytokine secretion, and enhanced migration/invasion capability.^[^ ^9^ ^]^ Acellular ECMs with attractive bionic spatial arrangement and biomechanical attribute have caught more attention to complement tumor microenvironment in tumor metastasis and recurrence.^[^ ^10^ ^]^ These evidences will suggest that exploring acellular natural matrices to serve as the tumor model will provide a new approach for tumor immune therapy and antitumor drug screening testing.

Polydopamine (PDA), a melanin‐like biopolymer, can be conveniently modified onto organic and inorganic substrates by oxidative polymerization of dopamine (DA) in alkaline condition.^[^ ^11^ ^]^ The PDA‐coating layer will present versatile and biocompatible features. More importantly, PDA‐based biomaterials can also play the paramount role on drug delivery carrier and polymer scaffold decoration, including bone or muscle regeneration, nerve formation, and vascular development.^[^ ^12^ ^]^ Therefore, PDA‐modified biofunctional 3D scaffolds have significant potential in the application of biomedical engineering and antitumor immune drug screening. To overcome the challenges of natural 3D scaffolds, in this study, a PDA‐functionalized 3D lung scaffold bioplatform was designed to construct complicated breast tumor microenvironment for anticancer drug screening and immunotherapy. The 3D organotypic tumor biosystem was synthesized by PDA modification of decellularized porcine lungs tissues and showed similar anatomical, histological, and physiological characteristics with human beings. After PDA modification, tumor scaffolds presented the excellent water absorption, hydrophilia, biocompatibility, and cell adhesion. Single‐cell suspension from tumor tissue was collected and cultured on the PDA‐modified scaffolds. CD45^+^/CD3^+^/CD4^+^/CD8^+^ T cells were alive for over 72 h and has potential to monitor the ability of antitumor immune drug screening. The mechanism of cell growth, proliferation, and response to different chemotherapeutic drugs was systematically studied on 3D PDA‐modified scaffolds. Therefore, the PDA‐modified scaffolds could provide a promising platform for immune‐oncology drug screening.

## 2 Results

### 2.1 Preparation and Characterization of 3D PDA‐Modified Scaffolds

**Figure 1:**
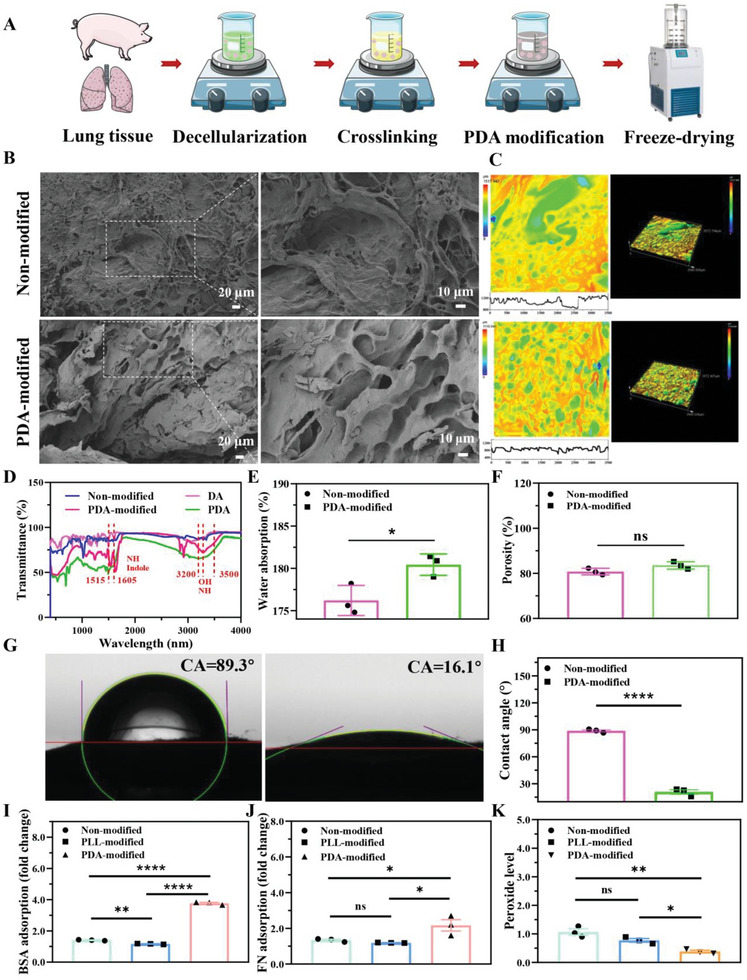
Preparation and characterization of PDA‐modified scaffold. A) The preparation process of PDA‐modified scaffold. B) Representative SEM images of PDA‐modified scaffold. Scale bar: 20 µm. Right side panel was amplifying SEM images. Scale bar: 10 µm. C) Surface images and 3D view of nonmodified and PDA‐modified scaffold by LSCM. D) FTIR analysis of DA, PDA, nonmodified, and PDA‐modified scaffold. E) Water absorption rate. F) Porosity of 3D scaffolds. G,H) Water contact angles of nonmodified and PDA‐modified scaffold. I) BSA absorption. J) FN absorption. K) Removal ability of peroxide in different scaffolds. After 2 h culture in PBS with 1 µm hydrogen peroxide, peroxide level was measured (*n* = 3, ns was no significance, *p < 0.05, **p < 0.01, ****p < 0.0001).

The 3D biofunctional scaffolds were prepared from fresh pig's lungs by a decellularized bronchi method and further enhanced for cell molecular adhesion via PDA modification. A schematic illustration of the scaffold fabrication process is shown in **Figure** **1**A. The 3D scaffold microstructures were measured to observe the geometric morphogenesis by scanning electron microscope (SEM). The 3D PDA‐modified scaffolds showed regularly rough topology with distinct pores (Figure 1B). It has been reported that porous structures are crucial for cell adhesion, migration, and proliferation.^[^ ^13^ ^]^ To further investigate surface morphology and uniformity of the scaffolds, a 3D measuring laser microscope was applied to evaluate the roughness before and after PDA modification. Comparing with the bulk uneven microtopography of PDA‐unmodified lung scaffolds, PDA‐modified scaffolds displayed homogenous, flat, and smooth morphological microstructures (Figure 1C). The roughness appearance showed the similar morphology with SEM observation. In addition, the gross presentation of lung scaffolds would be altered from white to black after PDA modification. These results suggested that PDA modification was beneficial for uniformly rough microstructures of 3D lung scaffolds.

The 3D scaffolds were further analyzed to investigate the chemical synthesis stability. As shown in Figure 1D, Fourier transform infrared (FTIR) curves of the nonmodified and PDA‐modified scaffolds both showed the curves (amide A, I, II, and III bands) of vibration of amide groups. Amide bands (I and II) shifted from 1634 to 1630 cm^−1^ and 1539 to 1548 cm^−1^ after Ethyl‐3‐(3‐dimethyl aminopropyl) carbodiimide (EDC)/N‐hydroxy‐succinimide (NHS) crosslinking reaction, respectively. The absorption band of the amide A band at 3286 cm^−1^ was responsible for the coupling of N─H stretching vibrations to hydrogen bonds. 1515 and 1605 cm^−1^ were the characteristic absorption peaks of PDA, consistent with the indole or indoline structure and vibration of ─NH_2_ group.^[^ ^14^ ^]^ The broad peaks between 3200 and 3500 cm^−1^ were related to hydroxyl and amine structures, with phenolic hydroxyl vibration peaks at 1345 and 1285 cm^−1^. The results showed that PDA was successfully grafted onto 3D lung scaffolds for tumor cell culture.

Porous 3D scaffolds were available to enhance cell permeability and migration behavior. The porosity in the PDA‐modified and nonmodified scaffolds was calculated to be 83.50±1.65% and 80.77±1.46%, respectively, which had little significant difference (Figure 1E). Merely, because of PDA intervention, the water absorption increased from 176.20 ±1.78% to 180.43 ±1.26% (*p* < 0.05) (Figure 1F). The water contact angle values were also measured to detect the surface wettability of post‐PDA modification (Figure 1G). The water contact angle (CA) of nonmodified scaffold was 89.3°±3.1°, while the angle of PDA‐modified scaffold surface was significantly reduced to approaching 0° (Figure 1H), indicating that slightly hydrophobic 3D scaffolds were transformed into superhydrophilic surfaces. Hydrophilic scaffolds were favorable for cell spreading and adhesion. In addition, the 3D PDA‐modified scaffolds could enhance bovine serum albumin (BSA) adsorption (Figure 1I) and fibronectin (FN) adsorption (Figure 1J) to acquire outstanding protein adsorption ability for cell attachment, yet decrease the peroxide level (Figure 1H) to remove peroxide residues for cell viability after coincubation. Therefore, the architecture of 3D PDA‐modified scaffolds were constructed through morphological observation, FTIR, porosity, and wettability analysis.

### 2.2 Cell Biocompatibility, Proliferation, and Spreading on 3D PDA‐Modified Scaffolds

**Figure 2:**
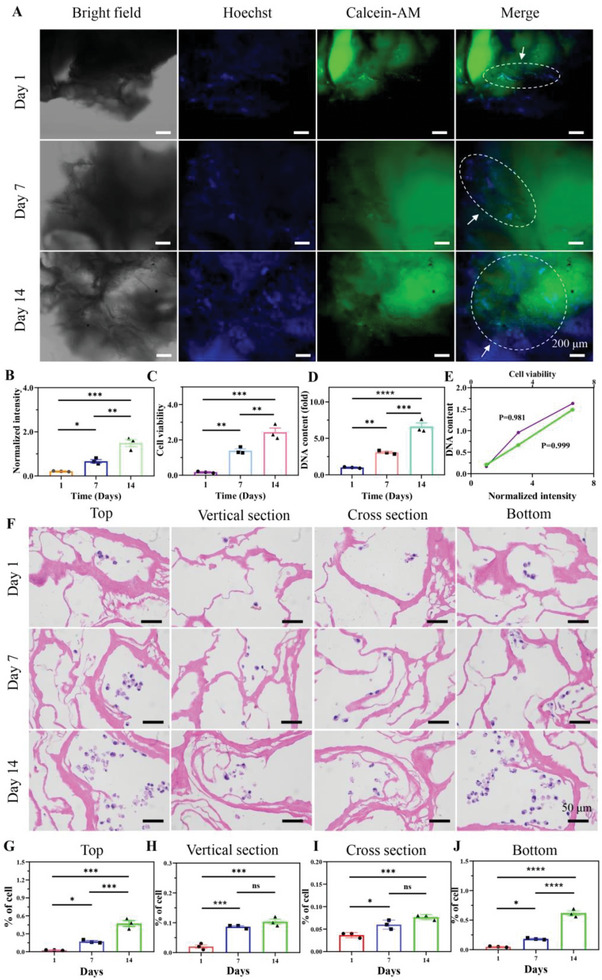
Growth and spreading of MCF‐7 cells on the PDA‐modified scaffold at days 1, 7, and 14. A) The MCF‐7 cells growth on the PDA‐modified scaffold by live/dead staining. Scale bar: 200 µm. B) Normalized intensity of live/dead staining. C) MCF‐7 cell viability at days 1, 7, and 14 on PDA‐modified scaffold. D) DNA content of MCF‐7 cells at days 1, 7, and 14 on PDA‐modified scaffold. E) Relationship of DNA content and normalized intensity and cell viability. F) Cell spreading in PDA‐modified scaffold was detected by HE staining. Scar bar: 50 µm. Cell number was amounted at top surface G), vertical section H), cross section I), and bottom surface J). The data present mean ± SD, *n* = 3. ns was no significance, *p < 0.05, **p < 0.01, ***p < 0.001, ****p < 0.0001.

Preferable cell compatibility is fundamental to build stable in vitro 3D tumor models.^[^ ^15^ ^]^ We determined the biocompatibility of the PDA‐modified scaffold by live staining, CCK‐8 assay, and hemolysis analysis, followed by analyzing the cell proliferation on the scaffolds. The cells on PDA‐modified scaffold were handled by live staining and the percentage of live cells was enhanced with increasing culture time from 1 to 14 days by amounting the number of live cells (**Figure** **2**A). Normalized fluorescence intensity was also calculated to present a gradually raised tendency with culture time increasing, indicating superior biocompatibility (Figure 2B). Nevertheless, the number of live cells and normalized fluorescence intensity on nonmodified scaffolds were lower than that in cooperation of PDA‐modified ones (Figure S1, Supporting Information). PDA decoration was favorable for enhancing cell adhesion and biocompatibility. Interestingly, cell viability was significantly doubled when cell culture time was elevated from 1 and 7 to 14 days (Figure 2C), showing good adaptability on PDA‐modified scaffolds. The cells could rapidly proliferate to construct a cell cluster with increasing time (Figure S2, Supporting Information). DNA content was measured to emerge a booming habit with culture time extending (Figure 2D). The content of DNA reached the maximum on day 14 to exhibit the excellent proliferation ability on 3D PDA‐modified scaffolds. Further, the proliferation ability displayed the great positive correlation with normalized intensity and cell viability by Pearson coefficient analysis (Figure 2E). In addition, blood compatibility is an essential factor for biocompatibility. The scaffolds were examined by hemolysis analysis (Figure S3, Supporting Information). Comparing with positive control (deionized water, DI), optical density (OD) values were decreased to around zero, showing significantly lower hemolysis rate for 3D scaffolds. Moreover, little statistically significant differences were observed between nonmodified and PDA‐modified scaffolds, which was considered biocompatible and safe for red blood cells. The quantitative results of fluorescent intensity, CCK‐8 assay and hemolysis analysis demonstrated that 3D PDA‐modified scaffolds performed the distinguished biocompatibility, low cytotoxicity, and attractive proliferation ability.

Further, the expression of extracellular matrix proteins and cell adhesion proteins were analyzed by immunofluorescence staining. The MCF‐7 cells attached and interacted with the PDA‐modified scaffold, which promoted the expression of some proteins (fibronectin, integrin, talin‐1, vinculin, β‐catenin, and actin filaments) related to cell anchoring junction (Figure S4A, Supporting Information). Fibronectin was expressed in ECM complexes to serve as the anchorage between cells and cell microenvironment (Figure S4B, Supporting Information). Transmembrane adhesion protein of integrin was formed to deliver these extracellular signals into cells by the regulation of integrin ligands, including talin, vinculin, and β‐catenin. These intracellular anchor proteins would interact with each other to form biophysical signaling bridge to alter cytoskeleton distribution, thereby mediating cell biological behaviors.

The cells were implanted on PDA‐modified scaffolds and observed at various time points (days 1, 7, and 14). After 14‐day culture, cell distribution was specifically assessed from different sections, including top, bottom, cross section, and vertical section by H&E staining (Figure 2F). The staining results revealed that cells completely infiltrated the entire PDA‐modified scaffolds. The growing MCF‐7 cells would spread and adhere on the porous frameworks and pores of the PDA‐modified scaffolds. The distribution of MCF‐7 cells on the scaffold showed a sandwich‐like spreading structure (Figure 2G–J). More cells would gather at peripheral region, while less cells assembled at central region, for example, the top (Figure 2G) and bottom (Figure 2J) slices had more cells, but there were fewer cells in the longitudinal section (Figure 2H) and central cross section (Figure 2I), which was due to a decrease in the oxygen gradient from the outside to the inside. Moreover, the number of cells was regularly enlarged with increasing culture time from 1 to 14 days, showing a similar results with living staining. Thus, the results suggested that cell biocompatibility, proliferation, spreading distribution could be well controlled on 3D PDA‐modified scaffolds.

### 2.3 Drug Resistance and Screening of Breast Cancer Cells on PDA‐Modified Scaffolds

**Figure 3:**
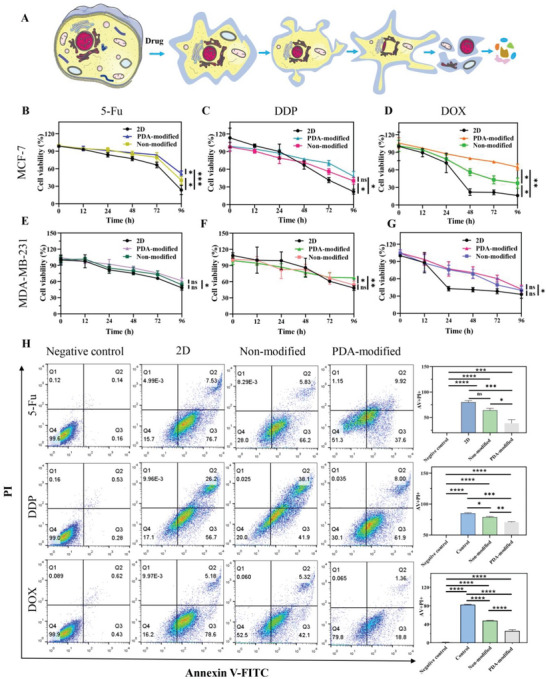
Increased 5‐FU, Cisplatin (DDP), and DOX drug resistance of breast tumor cells in PDA‐modified scaffolds. A) The process of cell apoptosis. The alternations included blebbing, cell shrinkage, nuclear deformation, chromatin condensation, DNA damage, and mRNA decay. Apoptosis results of 5‐Fu (B), DDP (C), DOX (D) for MCF‐7 cells and 5‐Fu (E), DDP (F), DOX (G) for MDA‐MB‐231 cells seeded on PDA‐modified samples with drug treatment for 96 h. The samples were analyzed by CCK‐8 assay. H) Flow cytometry results was conducted to investigate the influence of 5‐FU, DDP, and DOX on cell viability in 12‐well plate (2D), nonmodified scaffold (3D), and PDA‐modified scaffold after incubation for 96 h. The data present mean ± SD, *n* = 3. ns was no significance, *p < 0.05, **p < 0.01, ***p < 0.001, ****p < 0.0001.

Apoptosis is considered as a programmed cell death process by the proceeding of blebbing, cell contraction, nuclear fraction, condensation of chromatin, DNA disintegration, and RNA collapse to finally cause the end of cell fate.^[^ ^16^ ^]^ The 3D PDA‐modified scaffold was explored whether this bioplatform could better mimic the solid tumor concerning drug resistance and screening. We employed CCK‐8 and Annexin V‐FITC apoptosis detection assays to detect the effect of chemotherapy drug implementation on antitumor‐drug resistance and screening of 3D scaffolds in different culture condition (**Figure** **3**A). After the scaffolds were treated with respective chemotherapy drugs (5‐Fu, Cisplatin, and DOX), the apoptosis of all samples would be gradually promoted with culture time increasing while 3D PDA‐modified scaffold displayed the antitumor drug resistance and screening ability (Figure 3B–G). In specific, when the operation of 5‐Fu at 50 µg mL^−1^ for 96 h, 76.5%, 59.9%, and 48.4% of the cells underwent apoptosis at 2D culture, nonmodified scaffold, and PDA‐modified scaffold, respectively (Figure 3B). After the treatment of Cisplatin at 10 µg mL^−1^, the apoptosis rate was, respectively, 81.3%, 75.7%, and 69.5% on 3D PDA‐modified scaffold (Figure 3C). Further, DOX was applied in tumor cells at 50 µg mL^−1^ and the apoptosis rate was measured to be 83.8%, 62.7%, and 35.4%, respectively (Figure 3D). Moreover, MDA‐MB‐231 cells with strong invasiveness and high metastatic rate was also chosen as model breast cancer cell line for drug screening. After treatment with 5‐Fu at 50 µg mL^−1^ for 96 h, there was 50.6%, 54.0%, and 61.2% cells alive in 2D culture, nonmodified scaffold and PDA‐modified group (Figure 3E). After the treatment with Cisplatin (10 µg mL^−1^) and DOX (50 µg mL^−1^) for 96 h, the survival rate was, respectively, enhanced from 48.2% and 53.9% to 67.2% for Cisplatin addition (Figure 3F), and from 20.4% and 31.6% to 38.3% for DOX treatment (Figure 3G). There was an obviously increased cell viability rate in PDA‐modified group, compared with 2D culture and nonmodified group. Further, these results were also verified by the cell apoptosis analysis by flow cytometry (Figure 3H). It was noteworthy that tumor cells growing on a PDA‐modified scaffold showed lower sensitivity to chemotherapeutic drugs and stronger drug resistance, mimicking the solid tumor cell reactions for drug treatment better than 2D culture and nonmodified scaffolds. A possible assumption is due to enhanced cell contact and communication in compact 3D tumor cell clusters and elevated tight junctions that limit cell diffusion of drugs on PDA‐modified 3D scaffold.

### 2.4 Mechanism of Drug Resistance Screening of Tumor Cells on 3D PDA‐Modified Scaffolds

**Figure 4:**
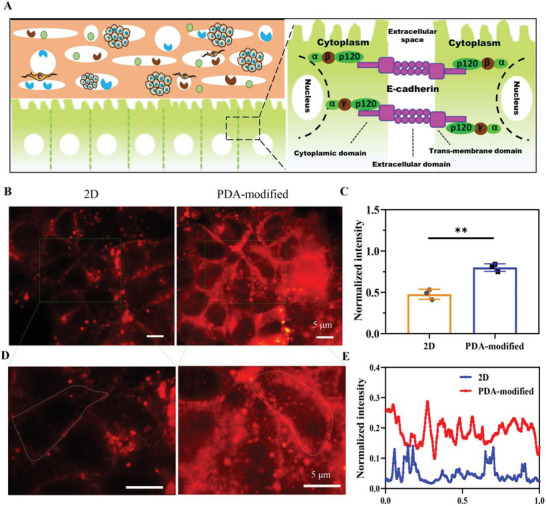
Mechanism of E‐cadherin expression of MCF‐7 cells in PDA‐modified scaffolds on drug resistance. A) Cell adhesion‐touching interaction via E‐cadherin formation. E‐cadherin is formed by inducing the adhesion of neighboring cells and associated with cell attachment and tight junction production. B) Immunolocalization of E‐cadhesion. They were detected using specific antibodies (Alexa Fluor 555 goat antirabbit IgG). Scale bar: 5 µm. C) Normalized intensity of stained E‐cadherin. D) Amplified images of stained E‐cadherin. Scale bar: 5 µm. E) Normalized intensity of white line, which represented the cell–cell junction. The data present mean ± SD, *n* = 3. **p < 0.01.

Principal considerations including cancer cell stemness, cell adhesion, cell proliferation, and DNA repair could contribute to drug chemoresistance during tumor treatment.^[^ ^17^ ^]^ The chemoresistance‐related markers, such as E‐cadherin, hypoxia, and cancer cell stemness were analyzed using immunofluorescence and qPCR further to explore the mechanism of drug resistance in MCF‐7 cells. First, the enhancement of cell contact and communication was regarded to be the main factor of drug resistance due to the elevated tight junctions. Cell contact and communication are closely related with survival, proliferation, differentiation, apoptosis, and even homeostasis.^[^ ^18^ ^]^ In this process, E‐cadherin would play the vital role in affecting cell junction formation and contact communication when cells attached on the PDA‐modified scaffold (**Figure** **4**A). We evaluated the E‐cadherin expression in breast tumor cells on 2D surface and PDA‐modified scaffold by immunofluorescence. The immunofluorescence results demonstrated that E‐cadherin labeling was formed on both 2D culture and PDA‐modified 3D scaffold, but more E‐cadherin was expressed on PDA‐modified 3D scaffold (Figure 4B). The normalized intensity of E‐cadherin was quantitatively measured and the fluorescent intensity of PDA‐modified scaffold was much stronger than that of 2D culture (Figure 4C). Notably, the fluorescent images were further amplified to display an intense, continuous, and complete cell contour at the periphery (Figure 4D). The peripheral E‐cadherin was analyzed by an ImageJ software and the results demonstrated that fluorescent intensity was strong at periphery to constitute an extended line due to cell contacts and communication (Figure 4E).

**Figure 5:**
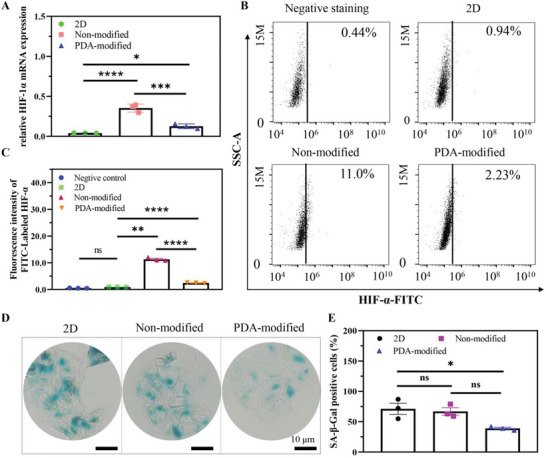
The hypoxia mechanism of drug resistance in MCF‐7 cancer cells seeded on PDA‐modified scaffold. A) The mRNA expression level of HIF‐1α. B) Flow cytometry images. C) Quantitative analysis of HIF 1α of breast cancer cells. D) Cellular senescence images. E) Quantitative analysis of MCF‐7 cells on different culture condition. Scale bar: 10 µm. The data present mean ± SD, *n* = 3. ns was no significance, *p < 0.05, **p < 0.01, ***p < 0.001, ****p < 0.0001.

Second, hypoxia is also a key factor to cause drug resistance.^[^ ^19^ ^]^ According to the results, hypoxia‐inducible factor‐1α (HIF‐1α) was accompanied with a higher mRNA expression in PDA‐modified group compared with 2D group, while lower HIF‐1α level than that of nonmodified group (**Figure** **5**A). Flow cytometry and its quantitative analysis also confirmed the upregulation of HIF‐1α in PDA‐modified group (Figure 5B,C). Upregulation of HIF‐1α induced by hypoxia‐mediated alterations in cell senescence has been reported to contribute to drug resistance.^[^ ^20^ ^]^ A HIF‐1α‐mediated decreased senescence was another underlying molecular mechanism. The staining results showed that MCF‐7 cells in PDA modified group would lead to a decreased senescence (Figure 5D) and the quantitative analysis revealed the similar tendency with staining images (Figure 5E), which demonstrated that decreased senescence was a crucial contributor to the development of hypoxia‐induced resistance.

**Figure 6:**
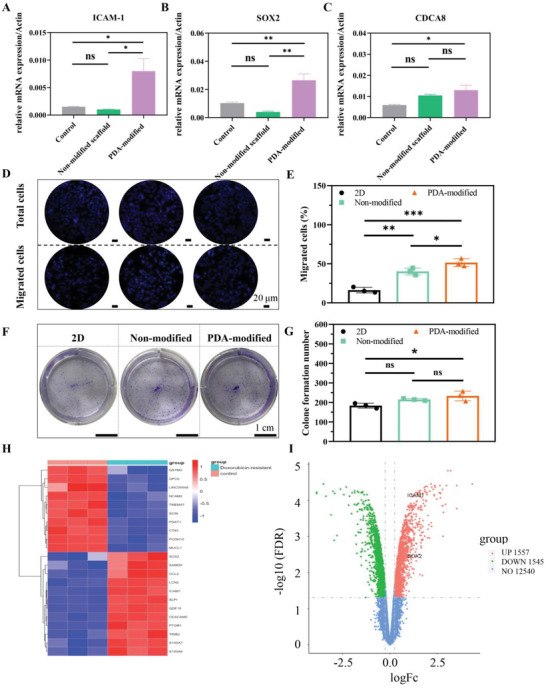
The stemness gene expression and the relationship with drug resistance. The mRNA expression of A) ICAM‐1, B) SOX2, and C) CDCA8. D,E) The migrated ability of MCF‐7 cancer cells at different conditions by transwell experiment. Scale bar: 20 µm. F,G) The invasive ability of cancer cells at different conditions by colon formation assay. Scale bar: 1 cm. H) The heat map and I) volcano plot of gene expression in breast cancer cells and DOX‐resistant MCF‐7 cells from GSE database. The data present mean ± SD, *n* = 3. ns was no significance, *p < 0.05, **p < 0.01, ***p < 0.001.

Third, HIF‐1α‐mediated upregulation of stemness genes could also confer drug resistance by the maintenance of cancer cell stemness on PDA‐modified 3D scaffold. Some reports have revealed that HIF‐1α can significantly upregulate pluripotent gene expression, such as SOX2, NONAG, OCT4, and KLF4 to restrain cell differentiation and keep the stemness phenotype.^[^ ^21^ ^]^ We evaluated the mRNA expression of stemness genes in all groups and we found the expression of ICAM‐1 (**Figure** **6**A), SOX2 (Figure 6B), and CDCA8 (Figure 6C) could be significantly upregulated in PDA‐modified group. Moreover, we analyzed the migratory and invasive level of MCF‐7 cells in all groups and the results demonstrated that cells cultured in PDA‐modified group presented higher migratory and invasive ability, which would contribute to drug resistance (Figure 6D–G). Therefore, drug resistance and screening could be regulated by the synergistic effect of E‐cadherin formation, hypoxia level, and stemness gene expression.

The ability of DNA damage repair in tumor cells was also one of the vital factors in drug resistance. O6‐methylguanine (O6‐MG)‐DNA‐methyltransferase (MGMT) was a strong drug‐resistance gene and became a target to protect hematopoietic stem cells during chemotherapy. MGMT expression related to DNA repair was remarkably higher in the 3D scaffold group and relatively higher in the PDA‐modified scaffold group, which indicated better DNA repair function of cells cultured on the PDA‐modified scaffold (Figure S5A, Supporting Information). In addition, DNA topoisomerases were the biofunctional enzymes to alter the DNA topology and served as the specific targets of anticancer drugs. The expression of topoisomerase 1 and 2 in MCF‐7 cells on the PDA‐modified scaffold was slightly higher than the control group (Figure S5B,C, Supporting Information). Furthermore, due to the significant correlation between enhanced expression of HIF‐1 and poor patient prognosis and tumor resistance, it is considered a target for antitumor drugs. To verify whether tumor resistance mechanisms were consistent in lung scaffolds and patients, we downloaded and analyzed the GSE data. The results revealed that there was a higher expression of SOX2 and ICAM‐1 in drug‐resistant group of patients, which was accordance with that in PDA‐modified group (Figure 6H,I). Moreover, previous studies showed that the pattern of gene expression in MCF‐7 cells on 3D scaffold was highly conformable to patient cancers with deficient prognosis. Therefore, tumor drug resistance mechanisms were consistent in lung scaffolds and patients.

### 2.5 Immune Cells on the PDA‐Modified Scaffold for Antitumor Immunotherapy

**Figure 7:**
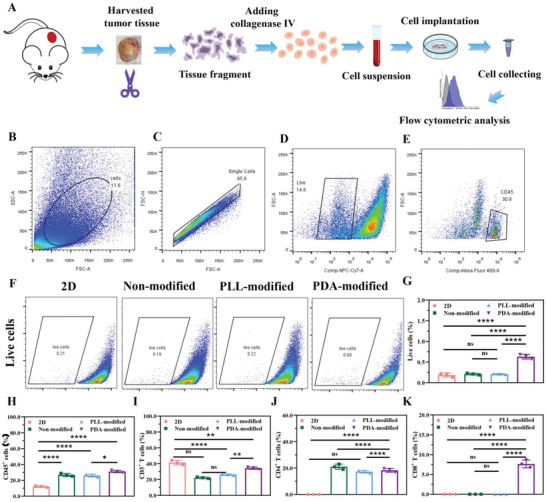
Drug screening of immune cells in single cell suspension cultured on PDA‐modified scaffold. A) The process of culturing single cell suspension on PDA‐modified scaffold. The steps were considered to harvest the dissociated tumors, digest into single cells and culture on PDA‐modified scaffold. The flow cytometry analysis of B) gated cells, C) single cells, D) live cells, and E) CD45^+^ T cells. After culturing for 72 h, F,G) live cells, H) CD45^+^ T cells, I) CD3^+^ T cells, J) CD4^+^ T cells, and K) CD8^+^ T cells. The data present mean ± SD, *n* = 3. ns was no significance, *p < 0.05, **p < 0.01, ****p < 0.0001.

The dissociated tumors were harvested from tumor tissue, digested into single cells, and cultured on a PDA‐modified scaffold to mimic the tumor microenvironment and investigate the immune cell state on scaffolds (**Figure** **7**A). After 48 h culture, the cells were analyzed with flow cytometry under being dyed with different markers. According to calculation, 11.6% of cells were gated (Figure 7B), of which 95.8% were single cells (Figure 7C). Of all single cells, 14.6% were viable (Figure 7D), from which 30.8% were CD45^+^ T cells (Figure 7E). These results revealed that the biofunctional PDA‐modified scaffolds might have broad application prospects in anticancer immunotherapy. After 72 h cell culture, the viability of the cells was greatly reduced (Figure 7F,G). CD45^+^ T and CD3^+^ T cells showed more numbers in PDA‐modified scaffolds than that in nonmodified ones (Figure 7H,I, Figure S6, Supporting Information). CD45 is the marker of all lymphocytes because CD45 served as lymphocyte common antigen and was related with receptor‐protein tyrosine phosphatase to produce in most leucocytes.^[^ ^22^ ^]^ However, CD45^+^ T cells could not fully represent the overall immune status in the tumor microenvironment. CD8^+^/CD4^+^ T cells may better reflect the immune microenvironment and it was also necessary characteristic for the immune related drug screening. To more accurately reflect the immune status, we cultured the single cell suspension on scaffolds for 72 h and evaluated the CD3^+^, CD4^+^, and CD8^+^ T cells in all groups. The results showed that there was little CD4^+^ and CD8^+^ T cells alive in 2D culture condition, 25.0% and 16.7% CD4^+^ T cells alive and no CD8^+^ T cells alive in nonmodified and PLL‐modified group (Figure 7J,K; and Figure S6, Supporting Information). Interestingly, there was 16.7% CD4^+^ T cells and 8.33% CD8^+^ T cells alive merely in PDA‐modified group. Therefore, PDA‐modified scaffold was more suitable for reflecting the immune status and screening immunotherapy drug.

### 2.6 PDA Inhibits Reactive Oxygen Species (ROS) Level on PDA‐Modified Scaffold

**Figure 8:**
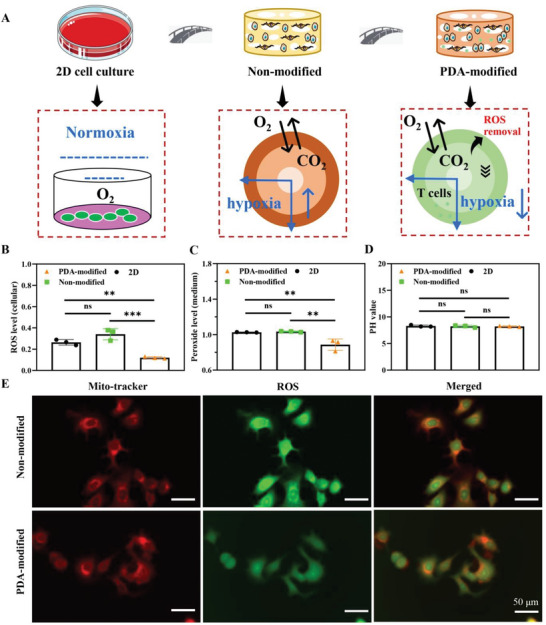
Influence of the PDA‐modified scaffold on ROS production and removal. A) The mechanism of PDA‐modified scaffold to be superior to 2D culture and PDA‐modified scaffold. In nonmodified scaffold, there was a hypoxia gradient and the hopoxia gradient decreased with ROS removal in PDA‐modified group. MCF‐7 cells from different groups were cultured, and the relative expression levels of total ROS B) and superoxide C) were measured by using a microplate reader. D) pH values of cell culture medium. E) ROS staining in live cells. ROS: green; mitochondria: red. Scale bar: 50 µm. The data present mean ± SD, *n* = 3. ns was no significance, **p < 0.01, ***p < 0.001.

**Figure 9:**
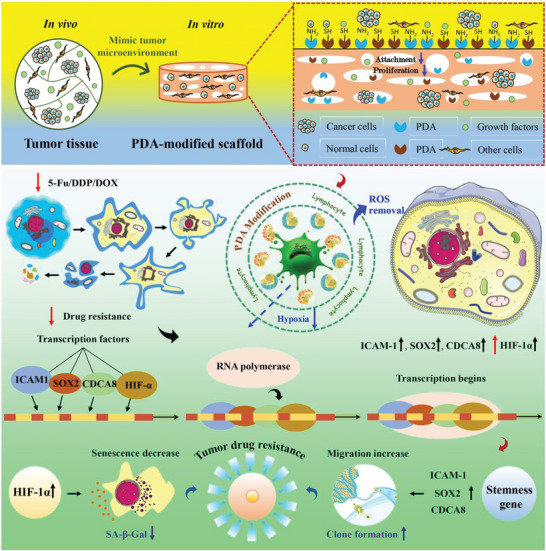
PDA‐functionalized 3D lung scaffold bioplatform to construct complicated breast tumor microenvironment for anticancer drug screening and immunotherapy.

Compared to the 2D group in normoxia, there was an oxygen gradient in nonmodified and PDA‐modified scaffold. In PDA‐modified group, there was a lower degree of hypoxia compared with nonmodified group, which provided a better oxygen environment for the survival of T cells. Therefore, the PDA‐modified scaffold could support a more dynamic and suitable microenvironment for the survival of tumor cells and T cells (**Figure** **8**A). After PDA modification, there was a typically lower level of entire ROS in cells by PDA regulation (Figure 8B). Furthermore, the quantity of peroxide was decreased in confined medium originating in MCF‐7 cells on PDA scaffolds (Figure 8C). However, the pH value of the medium did not be typically changed and the reason may be that the accumulation of ROS and peroxide was not enough to cause the alternation of pH value (Figure 8D). ROS is primarily formed in mitochondria to administer the function of tumor cells.^[^ ^23^ ^]^ We investigated ROS accumulation in mitochondria of MCF‐7 cells on different conditions. After 24 h culture, PDA‐modified group presented a lot lower ROS level in cells (green) than nonmodified group (Figure 8E). All in all, the observations revealed that PDA‐modified scaffolds diminished the oxidative stress in MCF‐7 cells through reducing ROS formation and eliminating the released and free ROS. The PDA‐modified scaffold with appropriate oxygen gradients and ROS levels could provide survival space for T cells to achieve the bright potential in antitumor immunotherapy. On the other hand, PDA‐modified scaffold could indicate antidrug resistance by 5‐Fu, Cisplatin, and DOX treatment for chemotherapy and drug resistance screening (**Figure** **9**).

## 3 Discussion

Tumor progression is closely related to the biomechanical and biomolecular cues of the tumor extracellular matrix (ECM).^[^ ^24^ ^]^ Tissue‐specific acellular matrix‐based platforms have gained increasing attention in the field of in vitro 3D tumor modeling due to their biomimetic properties to native tissue and ECM. Decellularized porcine lung has potential as a 3D tumor model because the larger surface area of the alveolar‐bronchioles in the scaffold can provide more binding sites for primitive cells to adhere and promote cancer cell proliferation and communication.^[^ ^25^ ^]^ In this study, we fabricated a 3D cancer model in vitro by decellularized porcine lung and PDA modification to investigate the influence of PDA modified 3D scaffold on antidrug resistance screening and immunotherapy in breast cancer model. Surface modification of biofunctional molecules is an excellent method to enhance cell adhesion and proliferation. Some studies have demonstrated that PDA could enhance cell division and differentiation by debilitating the thiol and amine group or growth molecules on PDA substrates.^[^ ^26^ ^]^ This study would successfully introduce PDA to modify the 3D scaffold. After PDA modification, the water contact angle value decreased to 16.1° and showed hydrophilic property. PDA has many hydrophilic groups (catechol and amine groups) (Figure S4, Supporting Information), which are favorable for the formation of hydrogen bonds, so the PDA‐modified scaffold has a lower water contact angle.^[^ ^27^ ^]^ The elevated hydrophilicity of the scaffold is vital for cell adhesion, which were under the ideal scaffold requirements. The PDA‐modified scaffold exhibited excellent biocompatibility, which demonstrated by MCF‐7 cells viability for 5 days. At 1 and 3 days, the OD values presented lower level in the PDA‐modified scaffolds than that in the 2D surfaces, which was due to the sufficient oxygen and nutrients in the first 3 days in the 2D culture (Figure S1C, Supporting Information). In 3D scaffolds, cells may experience hypoxic conditions and it will receive limited nutrients and oxygen than edge cells, consistent with a previous study.^[^ ^28^ ^]^ The results of cell attachment, spreading and proliferation proved that the PDA‐modified scaffold was more favorable for cell attachment and proliferation due to its improved hydrophilicity, biocompatibility, and suitable oxygen gradient. Moreover, PDA particles on the scaffold surface can provide protein adsorption sites to promote cell attachment and growth.^[^ ^29^ ^]^

The PDA‐modified scaffolds were also used to examine the effect of MCF‐7 cell drug resistance on different drug models. Our results showed that the PDA‐modified scaffolds increased MCF‐7 cells drug resistance to 5‐Fu, cisplatin, and DOX with elevated cell viability and decreased apoptosis rates. The E‐cadhesion formation, HIF‐1α‐mediated decreased senescence and improved tumor stemness were the main causes to drive drug resistance of breast cancer cells. Transmembrane protein E‐cadherin is considered as the key component of adherens junctions.^[^ ^30^ ^]^ It could combine EGFR signal subway and nuclear mechanotransduction to regulate polar tissue distribution and tight junction formation.^[^ ^31^ ^]^ It has been reported that the knockdown of E‐cadherin can promote tumor stemness‐like phenotype and antitumor drug resistance screening.^[^ ^32^ ^]^ HIF‐1α‐mediated pathway will also play an essential role in drug resistance and screening. Under the regulation of HIF‐1α, hypoxia‐mediated drug resistance to tumor therapy is correlated with reduced senescence.^[^ ^33^ ^]^ Moreover, drug resistance is closely associated with improved cancer cell stemness. Specifically, the high expression of various stemness genes (ICAM‐1, SOX‐2), a transcriptional regulator (CDCA8), and DNA repair‐related factors (MGMT), DNA topoisomerases 1 (TOPO1) were observed in PDA‐modified group. SOX2 overexpression increased breast cancer stem cells by activating the Wnt signaling pathway, resulting in drug resistance.^[^ ^34^ ^]^ Intercellular adhesion molecule‐1 (ICAM‐1) is considered as the transmembrane‐protein complexes, and its overexpression is a driving factor of breast cancer, as it participates in the recognition and adhesion between adjacent cells.^[^ ^35^ ^]^ Inhibition of ICAM‐1 expression could significantly reduce MCF‐7 cell drug resistance. Tumor cells cultured on ECM may trigger CDCA8 and promote drug resistance.^[^ ^36^ ^]^ TOPO1 and TOPO2 are important regulators of DNA replication and are overexpressed in cancer cells.^[^ ^37^ ^]^ The MGMT gene can also encode the typical protein of DNA damage repair to discard alkylating components for antidrug resistance of chemotherapy.^[^ ^38^ ^]^ A higher MGMT expression was exhibited in the nonmodified and PDA‐modified groups to achieve efficient drug resistance.

The development of tumor immunotherapy drugs and the prediction of therapeutic efficacy across all malignancies are urgently needed screening model to interrogate and perform anticancer immune response. The main obstacle is the availability of in vivo models to summarize the complexity of human malignant tumor and immune environment in tumor microenvironment.^[^ ^39^ ^]^ Currently, research on in vitro screening models for immunotherapeutic drugs is still difficult. To analyze the gap of trial system in the immune antidrug screening, a synthetic gel formulation was designed as the biofunctional scaffolds with increased tumor formation rate and decreased disease latency.^[^ ^4a^ ^]^ In our study, the PDA‐modified scaffold was constructed and showed a higher survival rate of CD45^+^ /CD8*^+^*/CD4^+^ T cells. Appropriate oxygen glucose and ROS are necessary for the survival of T cells.^[^ ^40^ ^]^ ROS can also induce activation‐mediated T cell apoptosis for cancer therapy.^[^ ^41^ ^]^ Therefore, PDA‐modified scaffold with ROS removal ability was more suitable for reflecting the immune status and screening immunotherapy drug. Our results was committed to establishing a biofunctional platform to accomplish economic and efficient antitumor immune drug screening for breast tumor treatment.

## 4 Conclusion

PDA‐modified scaffold based on acellular lung tissue were powerful in vitro platform. MCF‐7 cells attached and eventually spread on the scaffold. MCF‐7 cells cultured on the PDA‐modified scaffold showed drug resistance at the treatment of 5‐Fu, Cisplatin, and DOX. PDA‐modified scaffold showed better cell attachment property than 2D cell culture to mimic the in vivo cell states, which was an improved platform to study tumor progression. Changes in the related genes expression patterns led to the alteration in cell phenotype and function in the PDA‐modified group. Especially, HIF‐1α‐mediated alteration in cell division, survival, senescence, migration and invasion ability would contribute to drug resistance. The cell stemness, cell adhesion, cell proliferation, and DNA replication related gene expression in our 3D cell culture system was consistent with the primary tumor growing. Moreover, there was a higher survival rate of CD45^+^ T cells in PDA‐modified group. There was 16.7% CD4^+^ T cells and 8.33% CD8^+^ T cells alive only in PDA‐modified group. Therefore, PDA‐modified scaffold was more suitable to reflect the immune status and screening immunotherapy drug.

## 5 Experimental Section

### Preparation of PDA‐Functionalized 3D Decellularized Extracellular Matrices

All animal studies were approved by the Committee for the Ethics of Animal Experiments of Shanghai University (No. ECSHU 2021‐207) and in accordance with the Guidelines on the Care and Use of Laboratory Animals for biomedical research published by the National Institutes of Health (No. 85‐23, revised 1996). Fresh pork lungs were obtained from pigs (Luoyang Furun Meat Processing Co., LTD, China). After removing the blood and dissecting the bronchi, the small lung segments (8–12 cm^3^) were obtained. Then, to acquire the decellularized lung samples, these segments in 0.1% sodium dodecyl sulfate (SDS) for 1 day and 0.5% Triton X‐100 for 12 h were immersed. The segments were rinsed 60 min prior with phosphate buffer saline (PBS) and freeze‐dried in a lyophilizer. Subsequently, the samples were crosslinked for 6 h with 50 mm 1‐EDC/NHS in morpholine ethane sulfonate (MES) buffer solution and for further freeze‐drying. For PDA modification, the 3D scaffolds were immersed in tris base solution (pH = 8.5) with 2 mg mL^−1^ dopamine chloride (Sigma‐Aldrich, USA) for 6 h. Then, they were rinsed by PBS and for air‐drying.

### Characterization of PDA‐Functionalized 3D Decellularized Extracellular Matrices

The morphological structures of the lung scaffolds were characterized using SEM (JEOL, Japan). Images of the surfaces were captured by a laser scanning confocal microscope (OLS5000 LSCM, Japan). FTIR spectrophotometer (Mettler‐Toledo, Germany) was applied to analyze the chemicals and components of the scaffolds. The holder was placed in the groove of the sample plate and pressed for detection. The spectra of PDA‐modified and nonmodified scaffolds were observed in the range of 500–4000 cm^−1^.

### Water Absorption

The water absorption rate of the scaffolds was investigated using the gravimetric method. First, the weight of the tried scaffolds was calculated as the initial weight. Subsequently, the scaffolds were swollen in deionized water for 24 h to absorb water completely and then weighed. The water absorption ratio of the scaffold was computed as to the equation of Water Absorption Ratio = (*W*_wet_ *‐W*_dry_)/*W*_dry_×100%, where *W*_dry_ and *W*_wet_ were the sample weights before and after swelling, respectively.^[^ ^16^ ^]^

### Porosity Analysis

The scaffold's porosity was characterized using the apparent density method.^[^ ^17^ ^]^ First, samples were obtained and accurately measured its length, width, and thickness, and then calculated the volume (*V_i_*). After freeze‐drying, the weight of each sample (*m_i_*) was detected using an analytical balancer (0.1 mg), and the density (ρ_*i*_) of each scaffold was analyzed by the equation of *ρ_i_ = m_i_/v_i_*. The porosity (ε) was computaed from the measured average density (ρ) and the standard density of collegen ρ_0_( ρ_0_=  1.2±0.1 g cm^−3^). The porosity was characterized by equation of ε(%) *=* (1‐*ρ*/*ρ*_0_)×100%.

### Measurement of Surface Hydrophilicity

The drop shape analysis instrument (Biolin Scientific, Sweden) was applied to determine the water contact angle at ambient condition, where deionized water (6 µL) was dropped on the scaffold surface, and then the contact angle values were detected.

### Protein Adsorption Evaluation

The adsorption ability of proteins on 3D scaffolds was performed by BSA (bovine serum albumin, Sangon, China) and fibronectin (Sigma‐Aldrich, Germany) solution. A 100 µL amount of BSA solution with the concentration of 1 mg mL^−1^ in PBS or fibronectin solution with the concentration of 1 µg mL^−1^ in PBS was added in 96‐well plates with nonmodified, PLL‐modified, and PDA‐modified scaffold. After 2 h culture, the quantity of BSA was calculated by a BSA protein assay kit (Thermo Fisher, Germany). Fibronectin was amounted in the solution via a fibronectin ELISA kit (Thermo Fisher, Germany) by overnight culture. BSA and fibronectin were collected to analyze the quantity of BSA and fibronectin on the scaffolds.

### Hemolysis Analysis

A hemolysis assay was performed to investigate the blood compatibility of the scaffolds according to the ISO10993‐4 standard.^[^ ^42^ ^]^ Venous blood from healthy human volunteers was collected and kept them in anticoagulant tubes containing EDTA, diluted 4:5 by volume with normal saline. As the experimental group, the scaffolds were first immersed in normal saline for 30 min for the further use. Deionized water and PBS acted as the positive and negative control, respectively. Diluted blood (0.2 mL) was added to each group and maintained at 37 °C for 1 h. Then, the supernatant was collected by centrifugation at 3000 rpm for 5 min, and the OD value was measured at 545 nm. The hemolysis rate was obtained as follow

(1) $$\begin{array}{ccc} \mathrm{Hemolysis}\mathrm{ratio} & = & ({\mathrm{OD}}_{\left(\mathrm{test}\right)}-{\mathrm{OD}}_{\left(\mathrm{negative}\mathrm{control}\right)}/\\ & & \left({\mathrm{OD}}_{\left(\mathrm{positive}\mathrm{control}\right)}-{\mathrm{OD}}_{\left(\mathrm{negative}\mathrm{control}\right)}\right)\times 100\% \end{array}$$

### Cell Culture

The MCF‐7 and MDA‐MB‐231 cells were subcultured in the medium with 10% fetal bovine serum (FBS) and 1% penicillin‐streptomycin in 37 °C, 5% CO_2_ incubator. Before implanting cells, first, the scaffolds were sterilized by immersing the scaffolds in 75% ethanol for 8 h, soaking in PBS and then irradiating with UV for 2 h, then obtained the scaffolds. Subsequently, the scaffolds were air‐dried in a clean bench. 50 µL cell suspension (2 × 10^6^ cells mL^−1^) was seeded on the scaffold in a 24‐well plate. Dulbecco's modified eagle medium (DMEM) was added about 1 mL and replaced timely for subsequent experiments after incubation for 1 h.

### Biocompatibility

The scaffold's cytotoxicity was evaluated by the CCK‐8 assay and live/dead staining. 20 µL cell suspension (5.0×10^5^ cells mL^−1^) was implanted into scaffolds and 2D cell culture acted as a control. Cell viability was determined at days 1, 3, and 5 by adding CCK‐8 (10% v/v in DMEM). Then, it was kept at 37 °C for 4 h and measured the optical density (OD) at 450 nm. Moreover, at day 5, PDA‐modified scaffolds were stained in Calcein‐AM (2 µmol L^−1^), PI (10 µg mL^−1^), and Hoechst 33 342 solution (5 µg L^−1^) for 30 min. Nonmodified scaffolds were used as the control group. Subsequently, the cells were washed and captured with a fluorescent microscope (Nikon, Japan).

### Cell Proliferation

MCF‐7 cells on the PDA‐modified scaffolds were harvested and observed using a fluorescence microscope (Nikon, Japan) to observe cell proliferation in scaffolds. First, the samples were washed for three times and stained them with Hoechst 33 342 solution and Calcein‐AM solution (Solarbio, China) for 10 min. Subsequently, cell proliferation was imaged using a fluorescence microscope (Nikon, Japan).

### Cell Attachment and Spreading

Immunofluorescence and H&E staining was used to evaluate cell attachment and spreading. After culture, the scaffolds were treated by incubation with Fibronectin antibody (rabbit polyclonal antibody, 1:200), Vinculin antibody (rabbit monoclonal antibody, 1:200), β‐Catenin antibody (mouse monoclonal antibody, 1:200), Integrin β1 antibody (rabbit polyclonal antibody, 1:200), Talin‐1 antibody (rabbit polyclonal antibody, 1:200), F‐actin antibody (rabbit monoclonal antibody, 1:200) overnight. The scaffolds were treated with FITC conjugated goat antirabbit antibody (Beyotime, 1:50), and goat antimouse antibody (Beyotime, 1:50). Nuclei were contained with DAPI. The samples were fixed with 4% paraformaldehyde at day 1, 7, and 14. A 5 µm thick slice was sectioned from top, bottom, cross section, and vertical section and for H&E staining.

### Cell Migration and Matrigel Invasion Evaluation

For migration experiment, 10^4^ cells from monolayers or from 14‐day‐old PDA‐modified scaffold were put on the upper chamber of Boyden chambers in serum‐free DMEM medium, while the lower chamber was filled with FCS as the chemoattractant. 100 µL of Matrigel (1 mg mL^−1^) was precoated of the Boyden chamber filter. Then, Boyden chambers were cultured in 37 °C, CO_2_ incubator for 72 h and migration was determined. The upper cells were discarded by removing with a cotton swab. The cells that moved to lower region would be fixed and stained by DAPI to calculate the number. All assays were carried out in triplicate.

### Clone Formation

MCF‐7 cells in different groups were cultured with cell density of 7×10^2^ cells well^−1^ in 6‐well plate. After 14‐day culture, the colony growth was monitored and a colony was considered to be 50 cells or more. Then, the colony was fixed by 4% paraformaldehyde, stained by crystal violet staining solution (Beyotime, China) and counted using a microscope.

### ROS Detection

The intracellular complete ROS level were analyzed by a Reactive Oxygen Species Assay Kit (Beyotime, China). The cells were cultured in nonmodified and PDA‐modified scaffold for 3 days, followed by 2',7'‐Dichlorodihydrofluorescein diacetate (DCFH‐DA) staining in a warm incubator for 1 h. ROS fluorescent intensity was calculated by an Infinite 200 Pro microplate reader (Tecan Group Ltd., Switzerland). The mitochondria were contained by the MitoTracker kit (Beyotime Biotechnology, China). The fluorescent images were obtained by a confocal microscope (LSM780, Carl Zeiss, Germany). MCF‐7 cells were seeded on respective scaffolds. The peroxide concentration and pH value in the confined medium was calculated by Amplex Red Hydrogen Peroxide/Peroxidase Assay Kit (Thermo Fisher, Germany) and pH meter (Mettler toledo FE20, Switzerland).

To analyze the elimination ability of peroxide by PDA, the scaffolds were handled by using 1 µm hydrogen peroxide solution in PBS for 2 h to measure peroxide concentration in PBS.

### Cell Senescence Assay

MCF‐7 cells in 2D culture, nonmodified, and PDA‐modified samples were cultured at a density of 5.0×10^3^ cells well^−1^ of a 24‐well plate. After 14‐day incubation, the senescence‐associated β‐galactosidase (SA‐β‐Gal) was used to stain the samples and distinguish the senescent cells by a staining kit (Biotechnology, China). The fluorescent images were captured by a fluorescence microscope (Zeiss, Germany).

### Drug Response Analysis

5‐Fu (50 µg mL^−1^), Cisplatin (10 µg mL^−1^), and DOX (50 µg mL^−1^) were used as model drugs to detect cell sensitivity at different culture conditions. At first, MCF‐7 cells (5.0 × 10^5^ cells mL^−1^) were implanted in 24‐well culture plates, 3D scaffolds and PDA‐functionalized scaffolds, and cultured for 14 days, respectively. Then, the cells were handled with 5‐FU, Cisplatin, and DOX and the cell viability was detected by CCK‐8 experiment and Annexin V‐FITC/PI kit (Neobioscience, China) at various time points.

### Gene Expression Assay

RT‐qPCR was performed in a Biorad CFX Connected Real Time System (Biorad, USA) to evaluate the relative gene expression ability. First, according to a TRIZOL (Invitrogen, USA) method protocol, the total RNA was extracted from the cells on the scaffolds. Reverse transcription reactions were carried out using total extracted RNA (1.0 µg) by random hexanucleotides and a High‐Capacity cDNA Reverse Transcription Kit (Accurate Biology, China). 10 min at 25 °C, 120 min at 37 °C, and 5 s at 85 °C was the best thermal cycling condition. The final reaction volume was 20 µL, which included 1 µL cDNA, TaqMan Gene Expression Master Mix, and a TaqMan Gene Expression Assay (Applied Biosystems, USA). The thermal cycling condition was set as 30 s at 95 °C, 40 cycles of 5 s at 95 °C and 30 s at 60 °C. According to the Pfaffl formula, the relative gene expression of cells was calculated, which was defined as the proportion of targeting gene expression ability to endogenous gene expression level.^[^ ^19^ ^]^

### Flow Cytometry Analysis

The cell apoptosis state of MCF‐7 cells on scaffolds was carried out by an Annexin V‐FITC/PI assay kit (Neobioscience, China). The cells were collected and stained with Annexin V‐FITC and PI in binding buffer. For evaluating HIF‐1α expression, cells were treated by 2% formaldehyde, 0.1% Triton X‐100, and anti‐HIF‐1α (Cell signaling technology, USA) for 30 min away from light. For immune cells analysis, a single cell suspension (100 µL, 5×10^7^ cells mL^−1^) of dissociated tumor tissue was seeded on the scaffolds and cultured for 48 and 72 h. The cancer cells were harvested, rinsed with PBS, and resuspended. Then, the collected cells with Fixable viability stain 780, PerCP‐Cy5.5 Rat Antimouse CD45, FITC Hamster Antimouse CD3e, APC Rat Antimouse CD4, and PE Rat Antimouse CD8a (BD Biosciences, USA) were incubated at 4 °C for 30 min away from light. The tests were carried out on a FACS Celesta flow cytometer (BD Biosciences, USA) and calculated the data with FlowJo 10.

### Histology and Immunohistochemistry

The scaffolds were treated by 4% paraformaldehyde for 1 day. Then, these scaffolds in paraffin were embedded and cut them into 5 µm thick slices. The samples were obtained from paraffin‐embedded specimens for histological and immunohistochemical staining. After that, the samples in xylene were dewaxed and rehydrated them with graded industrial denatured alcohol. Finally, the sections were stained with hematoxylin and eosin, and then imaged.

### Statistical Analysis

The data were showed as the mean ± standard deviation (SD). One‐way ANOVA and using GraphPad prism8.0 (GraphPad, USA) was conducted for statistical analysis. *p* < 0.05 was identified as statistically significant (*). Each group was conducted three independent trials.

## Conflict of Interest

The authors declare no conflict of interest.

## Supporting information

Supporting Information

## Data Availability

The data that support the findings of this study are available from the corresponding author upon reasonable request.
