# Supplementary material for: A PDA‐Functionalized 3D Lung Scaffold Bioplatform to Construct Complicated Breast Tumor Microenvironment for Anticancer Drug Screening and Immunotherapy
Source: Adv Sci (Weinh). 2023 Jul 9;10(26):2302855. doi: 10.1002/advs.202302855 (PMC10502821; doi:10.1002/advs.202302855)
Supplement: Supplementary file 1 — Supporting Information [file ADVS-10-2302855-s001.pdf]

ADVANCED SCIENCE
Open Access

Supporting Information

for *Adv. Sci.*, DOI 10.1002/advs.202302855

A PDA-Functionalized 3D Lung Scaffold Bioplatform to Construct Complicated Breast Tumor Microenvironment for Anticancer Drug Screening and Immunotherapy

Wanheng Zhang, Yan Chen, Mengyuan Li, Shucheng Cao, Nana Wang, Yingjian Zhang and Yongtao Wang**

Supporting Information

A PDA-Functionalized 3D Lung Scaffold Bioplatform to Construct Complicated Breast Tumor Microenvironment for Anticancer Drug Screening and Immunotherapy

Wanheng Zhang[1,2,†], *Yan Chen*[2,†], *Mengyuan Li*[3,†], *Shucheng Cao*[4], *Nana Wang*[5], *Yingjian Zhang*[2,]*, *Yongtao Wang*[1,]*

[1]Shanghai Engineering Research Center of Organ Repair, School of Medicine, Shanghai University, Shanghai 200444, China

[2]Department of Pharmacy, the First Affiliated Hospital, and College of Clinical Medicine of Henan University of Science and Technology, Luoyang 471003, China

[3]School of Pharmacy, Nanjing University of Chinese Medicine, Nanjing 210023, China

[4]Department of Quantitative Life Sciences, McGill University, Montréal, Québec H3A 0G4, Canada

[5]Department of Pediatrics, Shanghai General Hospital, Shanghai Jiao Tong University, Shanghai 200080, China

[†]These authors contributed equally to this work.

[*]Correspondence author:

Dr. Yongtao Wang, E-mail: yongtao_wang@shu.edu.cn

Dr. Yingjian Zhang, E-mail: zbjhkd@sina.com

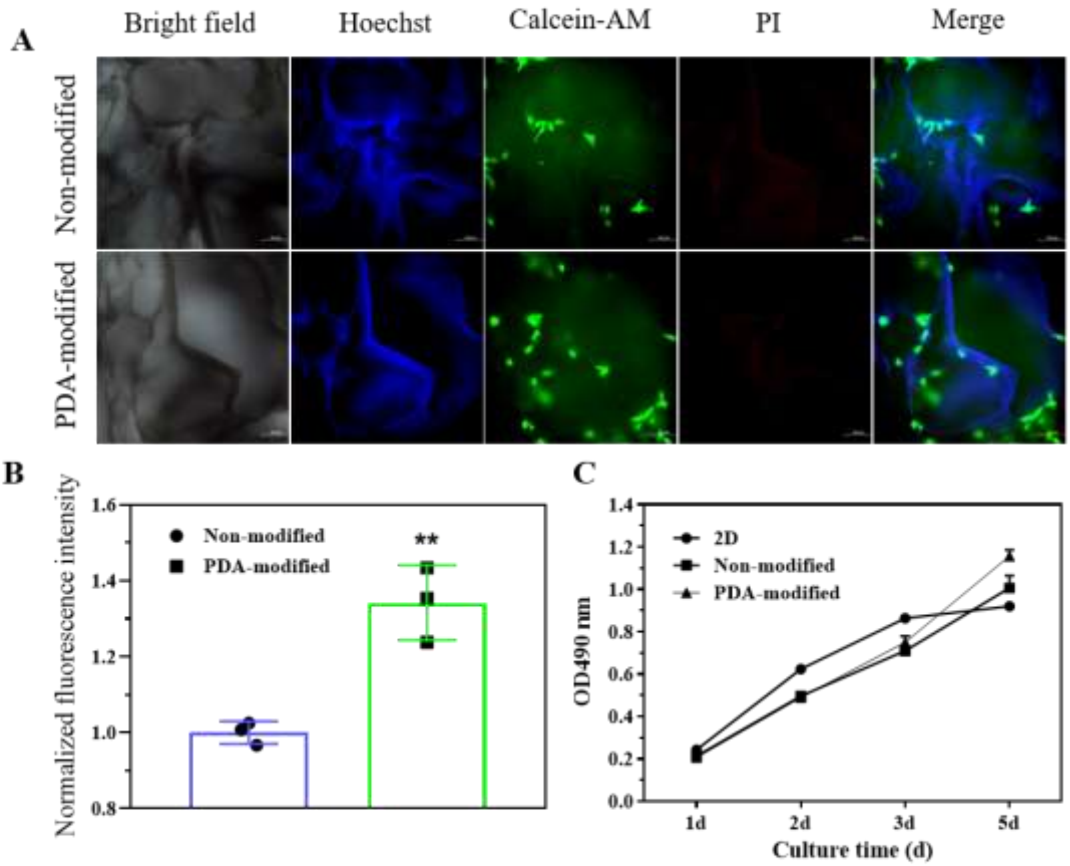

Figure S1. The biocompatibility of PDA-modified scaffold. (A) The cell viability observed by Calcein AM/PI and Hoechst staining for recellularization cultured in PDA modified and non-modified scaffolds at day 5. Scale bar: 100 μm. (B) The normalized intensity of stained cells. (C) The OD value of MCF-7 cells at different time points (day 1, day 2, day 3 and day 5).

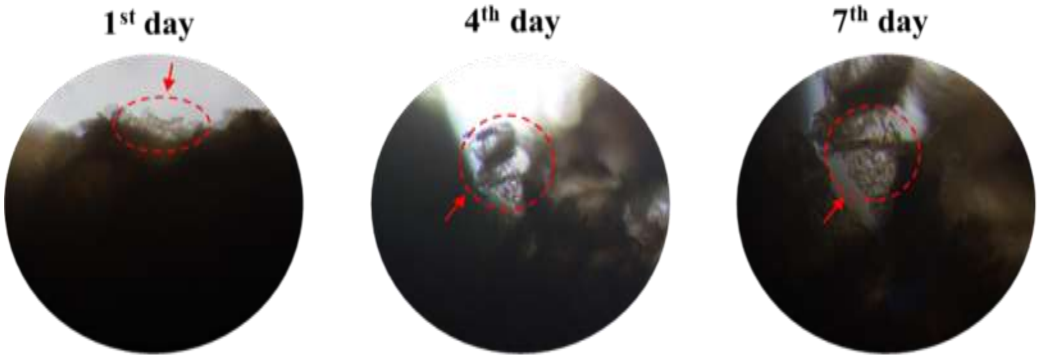

Figure S2. The cell cluster in different time point (day 1, day 4 and day 7) under bright field.

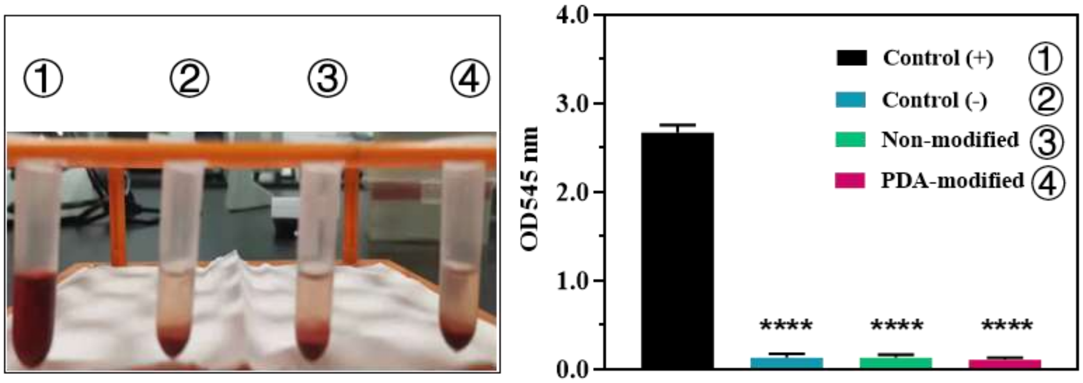

Figure S3. Hemolysis tests of the scaffolds. 3D scaffold and PDA-modified scaffolds are not hemolytic. Each value is mean ± standard deviation of triplicate determinations; n=3, ****p< 0.0001.

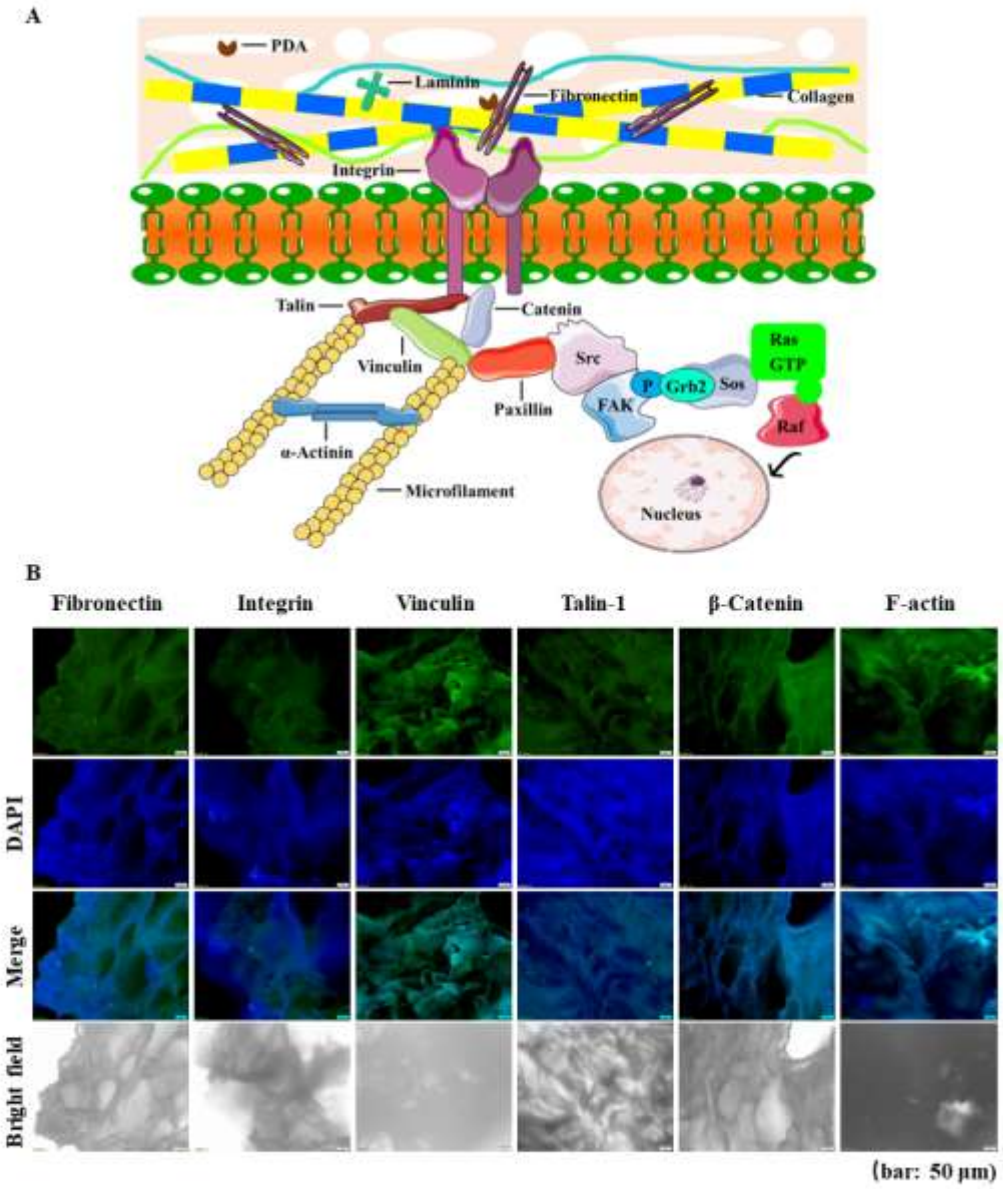

Figure S4. (A) Interaction of ECM proteins and intercellular adhesion proteins. (B) The immunofluorescence of Fibronectin, Integrin, Vinculin, Talin-1, β-Catenin and F-actin. Scale bar: 50 μm.

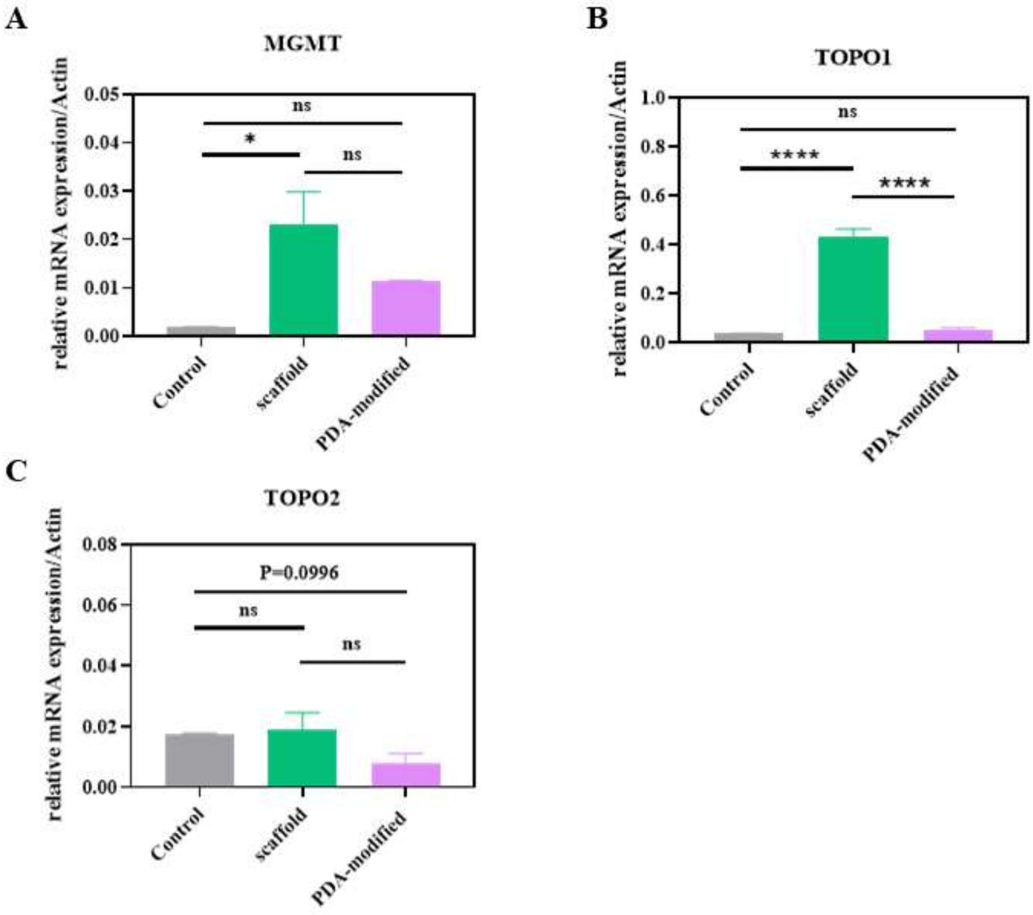

Figure S5. The mRNA expression of MGMT (A), TOPO1 (B), and TOPO2 (C).

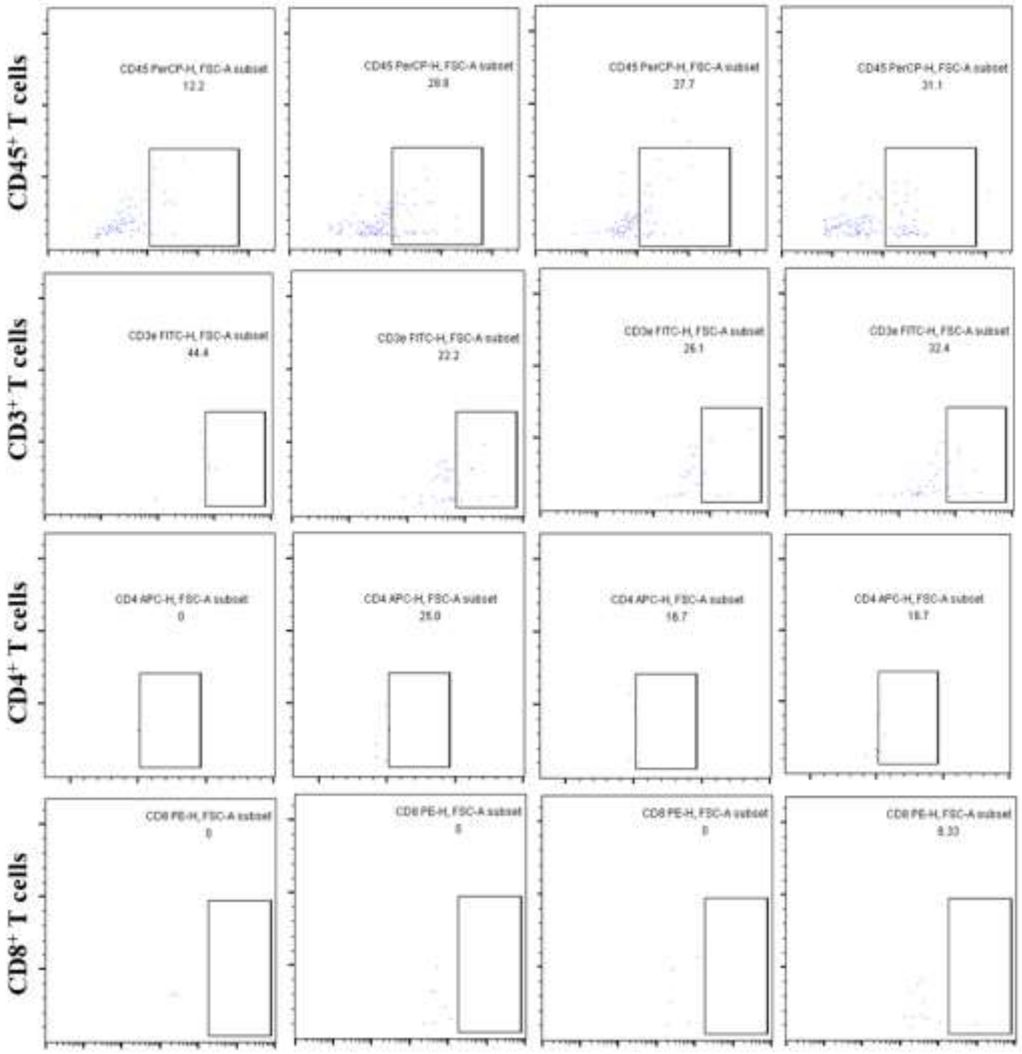

Figure S6. The T cells survival state cultured on the 2D, non-modified scaffolds, PLL-modified scaffolds and PDA-modified scaffold for 72 h.

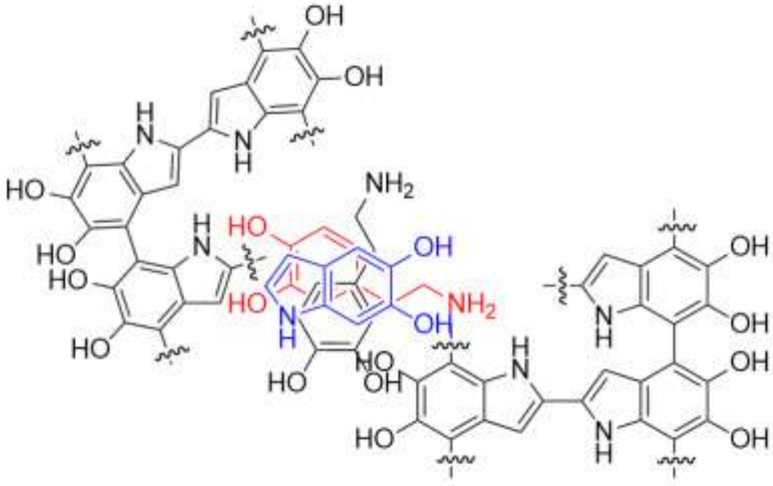

Figure S7. The structure of polydopamine.

Table S1. Sequences of the primers used in real-time qRT-PCR.

TOPO1 RT FP	AAGGTCCAGTATTTGCCCCAC
TOPO1 RT RP	ATTCATGGTCGAGCATTTTTGC
TOPO2 RT FP	GGTTCGTGTAGAGGGGTCAAG
TOPO2 RT RP	GCCGTCCACCTTTTGTAGTTG
ICAM1 FP	ATGCCCAGACATCTGTGTCC
ICAM1 RP	GGGGTCTCTATGCCCAACAA
CDCA8 FP	GCAGGAGAGCGGATTTACAAC
CDCA8 RP	CTGGGCAATACTGTGCCTCTG
SOX2 FP	GCTGCGAACAGTCAGACAGA
SOX2 RP	ACCTCCCGTCCAAGGTAGG
HIF α FP	GAACGTCGAAAAGAAAAGTCTCG
HIF α RP	CCTTATCAAGATGCGAACTCACA
MGMT FP	TTTTCCAGCAAGAGTCGTTCAC
MGMT RP	GGGACAGGATTGCCTCTCAT
MDR-1 FP	TGCCCCGCATATTCTCCCT
MDR-1 RP	CACCTGCGTTTTCGCTCTTG
